# Stereotactic Body Radiation Therapy for Stage I Non-Small Cell Lung Cancer: A Small Academic Hospital Experience

**DOI:** 10.3389/fonc.2014.00287

**Published:** 2014-10-20

**Authors:** Oren B. Factor, Charles C. Vu, Jeffrey G. Schneider, Matthew R. Witten, Scott L. Schubach, Alicia E. Gittleman, Donna T. Catell, Jonathan A. Haas

**Affiliations:** ^1^Division of Radiation Oncology, Winthrop-University Hospital, New York, NY, USA; ^2^Stony Brook School of Medicine, New York, NY, USA; ^3^Division of Medical Oncology, Winthrop-University Hospital, New York, NY, USA; ^4^Department of Thoracic and Cardiovascular Surgery, Winthrop-University Hospital, New York, NY, USA

**Keywords:** SBRT, non-small cell lung cancer, SABR, CyberKnife, local control, overall survival

## Abstract

**Purpose/Objective(s):** Stereotactic body radiation therapy (SBRT) has been shown to have increased local control and overall survival relative to conventional external beam radiation therapy in patients with medically inoperable stage I non-small cell lung cancer (NSCLC). Excellent rates of local control have been demonstrated both in clinical trials and in single-center studies at large academic institutions. However, there is limited data on the experiences of small academic hospitals with SBRT for stage I NSCLC. The purpose of this study is to report the local control and overall survival rates in patients treated with SBRT for stage I NSCLC at Winthrop-University Hospital (WUH), a small academic hospital.

**Materials/Methods:** This is a retrospective review of 78 stage I central and peripheral NSCLC tumors treated between December 2006 and July 2012 with SBRT at WUH. Treatment was given utilizing fiducials and a respiratory tracking system. If the fiducials were not trackable, a spine tracking system was used for tumor localization. CT-based planning was performed using the ray trace algorithm. Treatment was delivered over consecutive days to a median dose of 4800 cGy delivered in four fractions. The Kaplan–Meier method was used to calculate local control and overall survival.

**Results:** The median age was 78.5 years. Fifty-four percent of the patient population was female. Sixty seven percent of the tumors were stage IA, and 33% of the tumors were stage IB. Fifty-three percent of the tumors were adenocarcinomas and 29% were squamous cell carcinomas, with the remainder being of unknown histology or NSCLC, not otherwise specified The 2-year local control rate was 87%, and the 2-year overall survival was 68%.

**Conclusion:** Our findings support that local control and overall survival at a small academic hospital are comparable to that of larger academic institutions’ published experiences with SBRT for stage I NSCLC.

## Introduction

Surgery is the standard of care for stage I non-small cell lung cancer (NSCLC) (1) with local recurrence rates quoted around 5% (2, 3) and 5-year overall survival from 75 to 80% (4). In patients, who are medically inoperable or refuse surgery, radiation therapy is the next line treatment. Local control and overall survival with conventional radiation therapy have been demonstrated to be substantially inferior to surgery (5). Local control with conventional radiation therapy is reported from 40 to 70% with 5-year overall survival quoted from 7 to 32% (5).

Stereotactic body radiation therapy (SBRT) is becoming the standard of care in medically inoperable patients with stage I NSCLC (1). SBRT has been shown to be superior to conventional radiation therapy with local control from 83 to 93% and 3-year overall survival from 47 to 84.7% (4, 6–10). Most of the data from SBRT have come from clinical trials and single-center studies at large academic institutions. Data from smaller academic hospitals are more limited. The goal of this paper is to report the local recurrence rate and overall survival of patients with stage I NSCLC treated with SBRT at our institution. Additionally, this study performs an analysis of the patient, tumor, and treatment characteristics that predict local control and overall survival.

## Materials and Methods

### Patient population

This is a retrospective review of 78 stage I central and peripheral NSCLC tumors in 74 patients treated between December 2006 and July 2012 with SBRT at Winthrop-University Hospital (WUH). Peripheral tumors were defined at those >2 cm in all directions around the proximal bronchial tree. Patients who received CyberKnife for stage I NSCLC were identified from a WUH lung tumor registry. Patients were either medically inoperable or refused surgery. All patients were seen in conjunction with the Chairman of Thoracic Surgery to determine eligibility as to operability. It is primarily the decision of the Thoracic Surgeon who determines if a patient is a candidate for resection although the pulmonologist is often consulted. All patients had a PET/CT scan prior to treatment for staging purposes. As a general rule, PFTs were also obtained primarily to determine resectability. No patient had a FEV-1 <0.6 l. Patients who received chemotherapy or mediastinal radiation were excluded. All patients’ radiation oncology records were subsequently reviewed. This study was approved by our hospital’s institutional review board.

### Treatment technique

All 78 tumors were treated using a CyberKnife robotic linear accelerator. All patients were immobilized using a thermoplastic cast with arms up. One fiducial marker was placed at least 5 days prior either using CT guidance or navigational bronchoscopy to account for seed migration. CT imaging was performed using 1.5 mm cuts. Planning was performed using Multiplan (Accuray, Inc., Sunnyvale, CA, USA) inverse planning and delivered using the CyberKnife (Accuray, Inc.) with motion and respiratory tracking performed using the Synchrony system (Accuray, Inc.). For six tumors, the fiducials were not trackable, and a spine tracking system was used for tumor localization. The planning target volume (PTV) was created by adding a 5-mm margin to the GTV. In general, treatment was delivered over four consecutive days for central tumors to a dose of 4800 cGy delivered in four fractions (11). Peripheral tumors were treated to a dose of 6000 cGy in three consecutive fractions (6). The median dose delivered for all patients was 4800 cGy in four fractions. The various dose fractionation schemes are listed in Table 1. Patients treated with alternative dose fractionation schemes received prior radiation therapy. BED_10Gy_ was calculated for each of our dose schedules using the formula *nd*(1 + *d*/α/β), where *n* is the fractionation number, *d* is the daily dose, and α/β is assumed to be 10 for tumors. Tissue corrections and dose heterogeneity corrections were used in the treatment planning process. Dose constraints for normal tissues were the same as those used in RTOG 0236 (6).

**Table 1:** **Dose fractionation schemes**.

Dose	Number of patients	%
30 (15 × 2)	1	1
32 (8 × 4)	1	1
40 (10 × 4)	2	3
40 (8 × 5)	1	1
45 (15 × 3)	14	18
48 (12 × 4)	39	50
60 (15 × 4)	5	6
60 (20 × 3)	15	19

### Follow-up

The primary endpoints of this study were local control, overall survival, and toxicities. Recurrence-free survival was also evaluated. Local failure was defined as tumor recurrence within or immediately adjacent to the treated field. Local control was defined as the absence of local failure. Recurrence-free survival was defined as the time from the end of treatment to occurrence of a local failure, mediastinal relapse, distant relapse, or death. Patients were followed up periodically using either CT scans or combined PET/CT scans. The most recent CT scan, PET/CT scan, or time to local failure was noted. Overall survival was determined by the most recent follow-up or time to death. Follow-up was based primarily on the radiation oncology records. In addition, any supplemental notes from other departments sent to the radiation oncology department were also used. Overall survival was confirmed using the social security death index.

### Statistical analysis

Actuarial local control, overall survival, and recurrence-free survival were calculated using the Kaplan–Meier method. Univariate analysis was performed using Cox regression. Factors analyzed in the univariate analysis included BED_10Gy_, gender, age, and T-stage. A BED_10Gy_ cut off of 106 Gy was used in the univariate analysis because this was the median BED_10Gy_ in this study. All statistical analysis was done in SPSS 21 (IBM, Armonk, NY, USA). Toxicity was determined using the CTCAE 4.0 classification.

## Results

Demographic, tumor, and treatment characteristics are as follows: the median age was 78.5 years (range 56–93 years). Thirty-six tumors (46%) were in male patients and 42 tumors (54%) were in female patients. Thirty-four tumors (44%) were peripherally located and 44 tumors (56%) were centrally located. Forty-one tumors (53%) were adenocarcinoma, 23 tumors (29%) squamous cell carcinoma, 10 tumors (13%) NSCLC, not otherwise specified, and 4 tumors (5%) had unknown histology. Fifty-two tumors (67%) were T1 and 26 tumors (33%) were T2.

**Figure 1:**
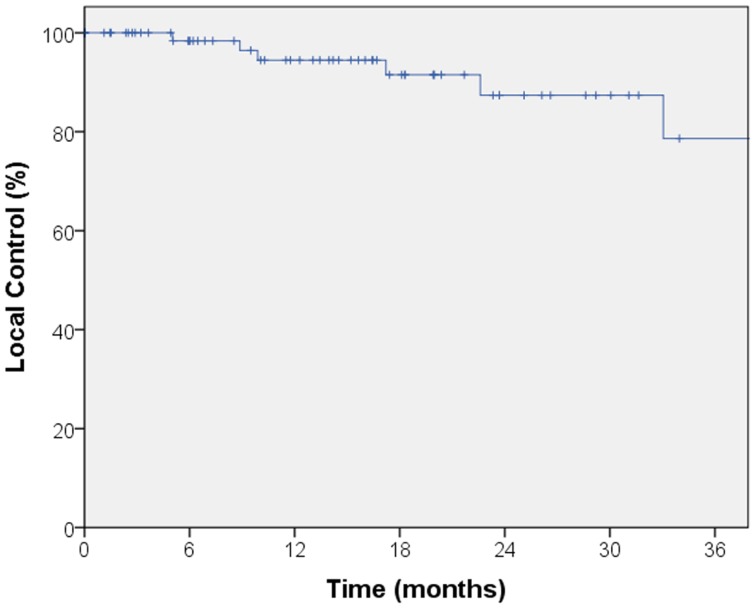
**Kaplan–Meier curve for local control**. The median follow-up for local control was 14.4 months. A total of seven local failures occurred in this study. The median time to local failure was 17 months with a range from 5 to 53 months. The 2-year local control rate was 87%.

**Figure 2:**
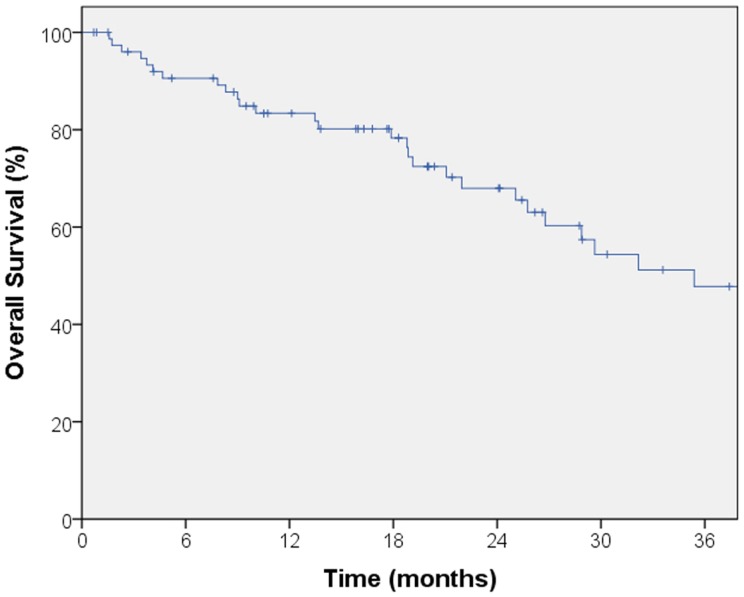
**Kaplan–Meier curve for overall survival**. Median follow-up for overall survival was 18.8 months. The 2-year overall survival was 68% in this patient population.

**Figure 3:**
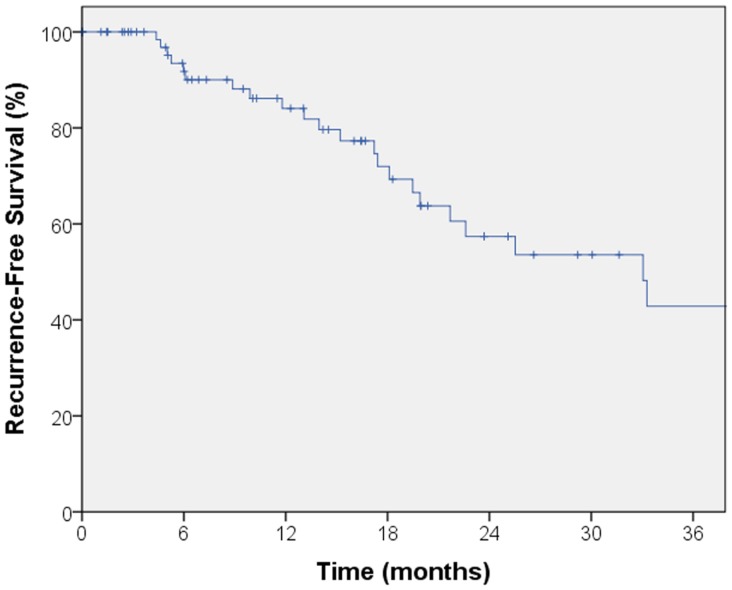
**Kaplan–Meier curve for recurrence-free survival**. There were 25 cases of recurrence of any form. The 2-year recurrence-free survival was 48.3%.

The median follow-up for local control was 14.4 months. A total of seven local failures occurred in this study. The median time to local failure was 17 months with a range from 5 to 53 months. The 2-year local control rate was 87% (Figure 1). Median follow-up for overall survival was 18.8 months. The 2-year overall survival was 68% in this patient population (Figure 2). There were 16 cases (22%) of mediastinal failure, and 10 cases (14%) of distant metastases. There were 25 cases (34%) of recurrence of any form. The 2-year recurrence-free survival was 48.3% (Figure 3).

Table 2 summarizes the univariate analysis of patient, tumor, and treatment characteristics on 2-year local recurrence and overall survival. Only male gender (*p* = 0.009) was associated with worse overall survival. Other patient, tumor, or treatment factors, including age, histology, T-stage, and biologic equivalent dose (BED_10Gy_), did not predict overall survival. No factors, including patient gender, were able to predict local recurrence.

**Table 2:** **Univariate analysis of predictors of local control and overall survival**.

		Local control		Overall survival	
		2 years LC (%)	*p*-Value	2 years OS (%)	*p*-Value
Gender	Male	78	0.23	57	0.009
	Female	92		77	
Age	<75	92	0.43	64	0.59
	≥75	85		70	
Histology	Adeno	95	0.54	74	0.2
	SCC	83		60	
TNM	T1N0M0	90	0.15	68	0.66
	T2N0M0	82		68	
BED (Gy_10_)	≤106 Gy	85	0.36	60	0.85
	>106 Gy	92		76	

There was one case of grade 2 pneumonitis identified. There were no reported cases of radiation pneumonitis greater than grade 2. There were no other toxicities experienced in this study.

## Discussion

Clinical trials as well as single-center studies have shown SBRT to provide far superior local control and overall survival when compared to conventional radiation therapy in the treatment of clinically inoperable stage I NSCLC, establishing SBRT as the standard of care in these patients (1, 6–10). The 2-year overall survival (68%) and 2-year local control (87%) in this study are similar to clinical trials and studies at larger academic institutions. Table 3 summarizes the results of a number of previous SBRT studies at large academic institutions and clinical trials.

**Table 3:** **Results of previous SBRT studies at large institutions and clinical trials**.

Study	Number of patients	Median follow-up (months)	3-year Local control	3-year Overall survival
Timmerman et al. (RTOG 0236) (6)	55	34.4	90.6%	55.8%
Baumann et al. (7)	57	34.0	92.0%	60.0%
Crabtree et al. (4)	151	23.4	90.0%	47.0%
Lagerwaard et al. (8)	177	LC-20.0	93.0%	84.7%
		OS-31.5	
Shibamoto et al. (9)	180	36.0	83.0%	69.0%
Fakiris et al. (10)	70	50.2	88.1%	42.7%
Present study	74	LC-14.4	87.0% (2-year)	68.0% (2-year)
		OS-18.8		

There are only a limited number of studies using CyberKnife in the treatment of stage I NSCLC. CyberKnife therapy appears to have similar success for local control and overall survival to other tools for delivering SBRT. The local control with CyberKnife therapy is reported from 85.8 to 100% and the overall survival is reported from 45 to 87% (12–16). A number of previous CyberKnife studies are included in Table 4. The study by Vahdat et al. included in Table 4 only included patients with stage IA NSCLC.

**Table 4:** **Results of previous CyberKnife studies**.

Study	Number of patients	Median follow-up (months)	2-year Local control	2-year Overall survival
Chen et al. (12)	40	44.0	91.0% (3-year)	45.0% (3-year)
Collins et al. (13)	20	25.0	100%	87.0%
van der Voort van Zyp et al. (14)	60 Gy-59	15.0	60 Gy-96.0%	62.0%-all patients
	45 Gy-11		45Gy-78.0%	
	70 Total	
Brown et al. (15)	31	27.5	85.8% (4.5-year)	83.5% (4.5-year)
Vahdat et al. (16)	20	43.0	95.0%	90.0%
Present study	74	LC-14.4	87.0%	68.0%
		OS-18.8		

Our study found comparable local control and overall survival to previous CyberKnife studies in the treatment of stage I NSCLC. Our study has a comparable or superior sample size to many of the previous CyberKnife studies.

The correlation between overall survival and gender is not unique to our study. In a single institutional prospective study of 177 patients with potentially operable stage I NSCLC, Lagerwaard et al. found female gender to have superior overall survival (*p* = 0.02) on multivariate analysis (8). Lagerwaard did not find a correlation between overall survival and any other patient, tumor, or treatment characteristic.

Similarly, in a multicenter study involving 180 stage I NSCLC patients, Shibamoto et al. found women to have an overall survival of 86% and men to have an overall survival of 67% (*p* = 0.031) (9). This study also found women as well as younger patients to have more favorable local recurrence rates. The *p*-value was 0.027 for women and 0.014 for age ≤76.

The link between female gender and superior overall survival does not appear to be unique to SBRT. A study by Wilsnivesky and Halm involving 18,967 elderly patients with stage I and II NSCLC divided patients into those receiving surgery, those receiving chemotherapy or radiation but not surgery, and untreated patients (17). Women were found to have better overall survival than men in all three groups (*p* < 0.001). On multivariate analysis women had better overall survival regardless of treatment type. Potential explanations for better overall survival of females included hormonal, genetic, and metabolic factors.

Other studies have demonstrated dose to correlate with local control and overall survival (18, 19). In a retrospective study involving 257 patients with stage I NSCLC at 14 institutions, Onishi et al. found those treated with a BED_10Gy_ of 100 Gy or more to have statistically significant superior local control and overall survival to patients receiving a BED_10Gy_ of <100 Gy (18). Olsen et al. also found improved local control and overall survival when treated with a BED_10Gy_ of >100 Gy in a single-center study of 130 patients with early-stage NSCLC (18).

In a study of 505 patients with early-stage NSCLC, Grills et al. found a BED_10Gy_ of 105 Gy or greater to predict local control (20). In this study, local recurrence was 15% in patients treated with a BED_10Gy_ of <105 Gy and 4% in patients receiving a BED_10Gy_ of 105 Gy or greater (*p* < 0.0001). In our study, a BED_10Gy_ of greater than or less than 106 likely failed to correlate with local control because of the smaller sample size of our study.

This study is limited by its relatively small sample size, short follow-up period, and retrospective design. As in the case of BED_10Gy_, it is possible that some of the variables may have achieved statistical significance with a larger sample size.

In conclusion, our study at a small academic hospital demonstrates effective local control and overall survival of patients with stage I NSCLC treated with SBRT. These results are encouraging for the use of CyberKnife SBRT in the treatment of stage I NSCLC outside of major academic institutions.

## Conflict of Interest Statement

Dr. Jonathan A. Haas has received speaker’s honoraria from Accuray, Inc., Sunnyvale, CA, USA. The other co-authors declare that the research was conducted in the absence of any commercial or financial relationships that could be construed as a potential conflict of interest.
